# Modal Properties of Photonic Crystal Cavities and Applications to Lasers

**DOI:** 10.3390/nano11113030

**Published:** 2021-11-12

**Authors:** Marco Saldutti, Meng Xiong, Evangelos Dimopoulos, Yi Yu, Mariangela Gioannini, Jesper Mørk

**Affiliations:** 1 DTU Fotonik, Technical University of Denmark, DK-2800 Kongens Lyngby, Denmark; menxi@fotonik.dtu.dk (M.X.); evadi@fotonik.dtu.dk (E.D.); yiyu@fotonik.dtu.dk (Y.Y.); jesm@fotonik.dtu.dk (J.M.); 2 NanoPhoton—Center for Nanophotonics, Technical University of Denmark, DK-2800 Kongens Lyngby, Denmark; 3 Department of Electronics and Telecommunications, Politecnico di Torino, IT-10129 Turin, Italy; mariangela.gioannini@polito.it

**Keywords:** photonic crystal(s), extreme dielectric confinement, light–matter interaction, line-defect cavities, nanolaser, microlaser, Bloch modes, Fano laser

## Abstract

Photonic crystal cavities enable strong light–matter interactions, with numerous applications, such as ultra-small and energy-efficient semiconductor lasers, enhanced nonlinearities and single-photon sources. This paper reviews the properties of the modes of photonic crystal cavities, with a special focus on line-defect cavities. In particular, it is shown how the fundamental resonant mode in line-defect cavities gradually turns from Fabry–Perot-like to distributed-feedback-like with increasing cavity size. This peculiar behavior is directly traced back to the properties of the guided Bloch modes. Photonic crystal cavities based on Fano interference are also covered. This type of cavity is realized through coupling of a line-defect waveguide with an adjacent nanocavity, with applications to Fano lasers and optical switches. Finally, emerging cavities for extreme dielectric confinement are covered. These cavities promise extremely strong light–matter interactions by realizing deep sub-wavelength mode size while keeping a high quality factor.

## 1. Introduction

A photonic crystal (PhC) [1] is the optical analogue of a solid-state crystal. Electrons in a crystal travel as waves through the periodic potential induced by the crystal atoms and exhibit energy band structures [2]. Similarly, a PhC is a periodic dielectric patterning on the scale of the photon wavelength. In these structures, the frequency and wavevector of photons are linked in a *photonic* band structure. Depending on the energy and propagation direction of photons, there may exist photonic band gaps. In these frequency ranges, photons backscattered by the periodic refractive index constructively interfere, and photon propagation is effectively inhibited.

This allows for tight confinement of light to defects in the periodic photonic crystal and makes such structures ideal platforms for optical cavities.

**Figure 1:**
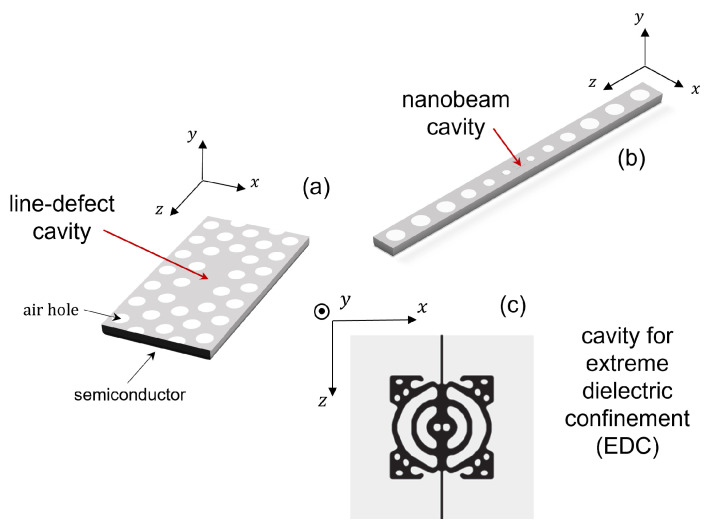
(**a**) Photonic crystal line-defect cavity, (**b**) photonic crystal nanobeam cavity and (**c**) a cavity for extreme dielectric confinement (EDC). In Figure 1c, the gray area is air and the black area is the semiconductor. Figure 1c is reprinted from [10], with the permission of AIP publishing, 2021.

In this work, we focus on photonic crystals consisting of air holes in a semiconductor material, rather than, e.g., isolated dielectric rods in air [1]. Figure 1 shows three examples of PhC cavities. A line-defect cavity (Figure 1a) is formed by introducing a defect within an otherwise (ideally) perfect PhC structure. This structure is carved out of a semiconductor slab (for instance, GaAs, InP or Si), by perforating the slab with a periodic patterning (i.e., a lattice) of air holes. The region surrounding the defect collectively acts as a distributed Bragg reflector, thereby efficiently trapping light inside the cavity. Light confinement in the vertical (i.e., *y*) direction is by total internal reflection. The hexagonal lattice ensures a large photonic band gap for TE polarization (i.e., with the electric field predominantly in *x* direction) [1] and is often employed in the realization of PhC lasers. In this case, the slab also includes the active medium, made of strained quantum wells [3,4] or quantum dots [5,6], which provide high gain for TE-polarized light. A cavity may also be realized in a semiconductor nanobeam [7] (Figure 1b). In this case, the photonic band gap confines the light in the longitudinal (i.e., *z*) direction, and confinement in the other directions is by total internal reflection. By exploiting a bow-tie structure, a cavity exhibiting spatial confinement of light deep below the wavelength of light in the material may be realized [8,9]. Figure 1c shows an example of such a cavity realized by topology optimization, which also displays such extreme dielectric confinement (EDC) [10].

### 1.1. Key Mode Characteristics: The Q-Factor and the Mode Volume

Broadly speaking, the strength of the light–matter interaction in a cavity increases when light is stored longer in the cavity and when the light is concentrated spatially, so that the intensity in the material increases.

The quality factor (Q-factor) reflects the temporal confinement of light. The Q-factor is defined as $Q=\omega_{0}U/P$ [11], where $\omega_{0}$ is the mode resonance angular frequency, *U* the electromagnetic energy stored inside the cavity and *P* the energy lost per unit time. By expressing *P* as dU/dt, one finds that the energy stored within the cavity decays with a time constant $\tau_{p}=Q/\omega_{0}$, which is the *photon lifetime*. It is customary to express the Q-factor as $Q^{-1}=Q_{c}^{-1}+Q_{i}^{-1}$. Here, $Q_{i}$ is the *intrinsic* or *unloaded* Q-factor, which accounts for radiation losses, and absorption losses due to impurities [12]. $Q_{c}$ reflects instead losses due to coupling with input and output waveguides. In the presence of a finite $Q_{c}$, the total Q-factor, *Q*, is often denoted the *loaded* Q-factor.

The spatial confinement of light is often quantified by a mode volume. The appropriate definition of such a mode volume depends on the type of light–matter interaction considered. For the Purcell-enhanced emission rate of a dipole emitter, it is customary to express the relevant mode volume as [13]

(1) $$V_{\mathrm{eff}}\left(r_{e}\right)=\frac{\int_{V}n^{2}\left(r\right){\left|E\left(r\right)\right|}^{2}d^{3}r}{n^{2}\left(r_{e}\right){\left|E\left(r_{e}\right)\right|}^{2}}$$

Here, *n* is the cavity refractive index, E the electric field and *V* the integration volume. Moreover, r is the position vector, with $r_{e}$ denoting the point where the emitter (e.g., a quantum dot) is placed. Therefore, for a given cavity, $V_{\mathrm{eff}}$ generally varies depending on the position of the emitter. If the emitter transition frequency coincides with the mode resonance frequency and the transition dipole moment of the emitter is aligned with the field polarization, the emitter decay rate within the cavity reads [14]

(2) $$\gamma_{r}=\frac{2d^{2}}{\hbar \epsilon_{0}n^{2}V_{\mathrm{eff}}\left(r_{e}\right)}\frac{\omega_{0}}{1/\tau_{p}+2/T_{2}}$$

where *d* is the transition dipole moment and $T_{2}$ the emitter dephasing time. The same emitter in a homogeneous medium with refractive index *n* would decay with a rate $\gamma_{r,\mathrm{hom}}=d^{2}\omega_{0}^{3}n/\left(3\pi \epsilon_{0}\hbar c^{3}\right)$ [14]. Thus, the emitter decay rate inside the cavity may be enhanced by a factor $F_{p}=\gamma_{r}/\gamma_{r,\mathrm{hom}}$—the Purcell factor [15]. This factor scales with $Q_{\mathrm{eff}}/V_{\mathrm{eff}}\left(r_{e}\right)$, where $Q_{\mathrm{eff}}=\omega_{0}{\left(1/\tau_{p}+2/T_{2}\right)}^{-1}$ is an effective Q-factor including the effect of the emitter and cavity broadening. Equation (1) is also widely employed as a loose figure of merit of the light spatial confinement. In this case, $r_{e}$ is often taken at an antinode, $r_{\max}$, of the cavity field.

Notice that the optical modes of a cavity with finite Q-factor are intrinsically leaky. Consequently, they diverge in space at sufficiently large distances from the cavity [16]. As a result, the integral in the numerator of Equation (1) diverges as the integration volume increases [17]. For this reason, a rigorous and unambiguous definition of the mode volume entering the Purcell-enhanced emission rate is that based on the theory of quasinormal modes [17,18]. However, the large Q-factor of PhC cavities makes the cavity modes slowly divergent [17]. In this case, Equation (1), with suitable integration boundaries enclosing the cavity, is often a good approximation. We emphasize, though, that one should also specify the integration volume, *V*, when employing Equation (1) [19]. Unfortunately, this is not always the case within the literature.

In the case of many emitters or nonlinear effects in bulk media, other mode volumes reflect the scaling of the light–matter interaction. Specifically, mode volumes relevant for nonlinear interactions are [20,21]

(3) $$V_{\mathrm{Kerr},\mathrm{TPA}}=\frac{{\left(\int_{V}n^{2}\left(r\right){\left|E\left(r\right)\right|}^{2}d^{3}r\right)}^{2}}{\int_{V_{\mathrm{nl}}}n^{4}\left(r\right){\left|E\left(r\right)\right|}^{4}d^{3}r},\;V_{\mathrm{FCA}}={\left[\frac{{\left(\int_{V}n^{2}\left(r\right){\left|E\left(r\right)\right|}^{2}d^{3}r\right)}^{3}}{\int_{V_{\mathrm{nl}}}n^{6}\left(r\right){\left|E\left(r\right)\right|}^{6}d^{3}r}\right]}^{\frac{1}{2}}$$

where $V_{\mathrm{Kerr},\mathrm{TPA}}$ ($V_{\mathrm{FCA}}$) is associated with the optical Kerr effect and two-photon absorption (free-carrier absorption). Here, $V_{\mathrm{nl}}$ is the volume of the nonlinear material. These mode volumes are usually larger than $V_{\mathrm{eff}}\left(r_{\max}\right)$ [22].

Finally, it should be noted that in the case of semiconductor lasers, the mode volume is conventionally defined as $V_{p}=V_{\mathrm{act}}/\Gamma$. Here, $V_{\mathrm{act}}$ is the physical volume of the active region, and Γ is the optical confinement factor, which roughly corresponds to the fraction of electric field energy stored inside the active region volume [23,24]

(4) $$\Gamma =\frac{\int_{V_{\mathrm{act}}}{\left|E\left(r\right)\right|}^{2}d^{3}r}{\int_{V}{\left|E\left(r\right)\right|}^{2}d^{3}r}$$

If the active medium consists of layers of quantum dots, the optical confinement factor per active layer is often computed as if the quantum dot medium were entirely filling the layer with thickness equal to the average thickness of the quantum dots [25]. This is correct as long as the filling factor of the quantum dot layer is accounted for in the expression of the material gain [25].

### 1.2. The Structure of This Paper

Applications of PhC cavities include PhC lasers, which are promising sources for optical interconnects [26], since they feature small footprint, low threshold current and reduced energy cost. As a result of the enhanced light–matter interaction, devices based on optical nonlinearities are also more energy-efficient [27,28]. Sub-femtojoule PhC switches have been demonstrated [29], as have PhC memories with ultralow power consumption in the nanowatt range [30] and large-scale integration [31]. Other applications include sensors with improved detection sensitivity [32], on-chip photodetectors with high quantum efficiency [33] and quantum cavity electrodynamics [34,35]. The high Q-factor of PhC cavities also enables adiabatic frequency conversion [36,37]. This phenomenon suggests a way to store and release photons on demand, with arbitrary timing. For thorough reviews of the many possible applications of PhC cavities, we refer to [22,28]. Specifically, excellent reviews of PhC lasers can be found in [38,39].

This paper is structured as follows. In Section 2, we give an overview of PhC cavities. In Section 3, we discuss threshold current and energy cost of microcavity lasers, such as PhC lasers. In Section 4, we cover the modal properties of passive, line-defect PhC cavities. In particular, we model a PhC cavity as an effective Fabry–Perot (FP) resonator, whose travel=ling modes are the *Bloch* modes of the waveguide on which the cavity is based. By this approach, we derive compact and transparent expressions for the resonance condition and field distribution. Furthermore, we discuss the impact of slow-light on threshold gain and out-coupling efficiency of line-defect PhC lasers. In Section 5, we review a new kind of PhC laser, known as the Fano laser [40], with a special focus on the tuning characteristics. In Section 6, we finally discuss cavities for extreme dielectric confinement, which promise even superior light–matter interaction than conventional PhC cavities.

## 2. Fundamental Principles of Light Confinement in Photonic Crystal Structures

**Figure 2:**
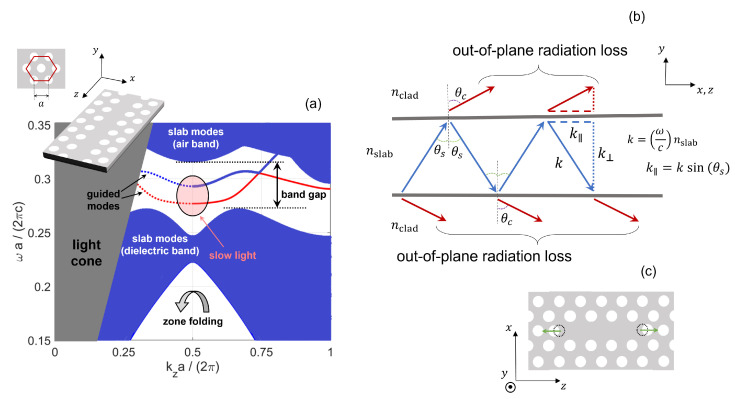
(**a**) A dispersion diagram of TE-like modes of a line-defect waveguide in a photonic crystal membrane, as depicted in the inset. The *y*-axis shows the angular frequency, ω, normalized to 2πc/a, with *c* being the light speed in vacuum and *a* the lattice constant. The *x*-axis shows the wavevector along the propagation direction, $k_{z}$, normalized to 2π/a. Modes in the gray area (the so-called light cone) are not confined by total internal reflection in the membrane. Details on the simulation parameters are provided in Section 4.1. (**b**) A ray-optics illustration within the light cone of the reflection and transmission at the interface between semiconductor slab and air cladding. (**c**) Optimization of a line-defect cavity by displacement of the holes at the edges of the cavity. The hole shift reduces the out-of-place radiation loss.

It is well known that defects in the crystalline structure of semiconductor materials (such as doping atoms) introduce additional states within the *electronic* band gap [41]. Similarly, defects in the periodic refractive index pattern of a PhC structure lead to additional states within the photonic band gap [42]. For instance, one may start from a semiconductor slab with an hexagonal lattice of air holes. By removing an entire row of holes, a line-defect is introduced. Thus, one creates a waveguide (see the inset of Figure 2a), where light is efficiently guided. Figure 2a shows the projected [1] band structure of a line-defect waveguide, with the cladding made of air. The band structure has been computed by the plane wave expansion method [43]. Further details on the simulation parameters and procedure are provided in Section 4.1. The *y*-axis displays the normalized angular frequency. The *x*-axis shows the normalized wavevector along the propagation direction, spanning over the right and left-hand sides of the first and second Brillouin zone, respectively. The line-defect introduces two bands within the band gap, corresponding to *guided modes*. These modes are confined in the vertical direction by total internal reflection, and confinement in the lateral direction is due to distributed Bragg reflection. Theoretically, these modes are truly lossless. Out-of-plane radiation losses only occur due to disorder [44], i.e., unavoidable fabrication imperfections. At the upper limit of the first Brillouin zone, the wavevector is equal to π/a, which is often denoted as the *band edge* and corresponds to the cutoff frequency of the guided modes. Close to the band edge, *slow-light* [45,46] may be achieved. In the slow-light propagation regime, the group velocity is much smaller than the light speed in vacuum and ideally tends to zero at the band edge. Other regions of the band structure include slab modes and the light cone. The *slab modes* are a continuum of modes which are confined along the vertical direction by total internal reflection, but are delocalized in the lateral direction. Conventionally, the lower-frequency (higher-frequency) band is denoted as dielectric (air) band, because lower-frequency modes in PhCs tend to concentrate their energy in the high refractive index regions [1]. These bands are analogous to the valence and conduction band of the electronic band structure of semiconductor materials. Finally, the *light cone* corresponds to modes which do not fulfill the condition of total internal reflection. These modes leak out of the semiconductor–air interface.

By removing a finite number, *N*, of air holes from a given row in a PhC semiconductor slab, a so-called line-defect cavity or LN cavity [47] is created, as shown in Figure 1a. The mode confinement in the longitudinal direction occurs by distributed Bragg reflection. Essentially, the mode guided along the line-defect lies within the photonic band gap of the perfectly periodic crystal stretching to the left and right of the cavity. Therefore, these periodic regions act as mirrors, between which the guided mode bounces back and forth. In contrast to line-defect waveguides, line-defect cavities suffer from out-of-plane radiation loss even in the absence of disorder. The spatial localization of the cavity mode thus gives rise to spatial frequencies, i.e., *k*-values, within the light cone. At a given frequency, the time-averaged power radiated out of the cavity is proportional to the integral within the light cone of the *spatial* Fourier transform of the electric field intensity [48]. Therefore, suppressing the wavevector components of the field within the light cone is an effective strategy to maximize the Q-factor.

The concept of light cone is better illustrated by Figure 2b. The Fourier transform of the electric field spatial profile consists of a set of plane waves, each with a given wavevector k. The light cone is determined by the component of k which is *tangential* to the interface between slab and cladding [49]. We denote the magnitude of this component by $k_{\|}$, with $k_{\|}^{2}=k_{x}^{2}+k_{z}^{2}$. If $k_{\|}$ cannot be conserved upon transmission through the interface, total internal reflection occurs. Otherwise, the plane wave with that wavevector can leak out of the slab. This is only possible if the angle of incidence, $\theta_{s}$ (see Figure 2b), is *smaller* than the critical angle $\theta_{s,cr}=\sin^{-1}\left(n_{\mathrm{clad}}/n_{\mathrm{slab}}\right)$. Here, $n_{\mathrm{clad}}$ and $n_{\mathrm{slab}}$ are the refractive indices of the cladding and semiconductor slab, respectively. As a consequence, the light cone corresponds to the region in the reciprocal space defined by $k_{\|}<\left(\omega_{0}/c\right)n_{\mathrm{clad}}$, with $\omega_{0}$ being the angular frequency of the resonant mode.

The rule of thumb to maximize the Q-factor is that the spatial envelope of the field should vary as smoothly as possible at the edges of the cavity [49]. Intuitively, this can be better understood by assuming the cavity to be one-dimensional. In this case, the field of the resonant mode in real space may be expressed as the product of an envelope and a sinusoidal wave, with spatial period given by the guided resonant wavelength. Therefore, abrupt changes in the envelope lead to high frequency components in its spatial Fourier transform. These components are then transferred to the light cone upon convolution of the envelope Fourier transform with the Fourier transform of the sinusoidal wave, thereby degrading the Q-factor. As a consequence, these abrupt changes should be avoided [12]. When the spatial extent of the cavity is decreased, the mode extends further in reciprocal space, with a considerable fraction of wavevector components lying within the light cone. Consequently, if the cavity is not properly designed, the out-of-plane radiation loss can severely limit the Q-factor. By displacing the holes at the edges of the cavity, the fraction of wavevector components within the light cone can be significantly reduced and the Q-factor thereby increased. This optimization procedure is illustrated by Figure 2c. Indeed, by displacing the holes, the periodicity of the crystal on either side of the cavity is perturbed and its reflection somehow weakened. Therefore, the field penetrates deeper within the crystal and is more gently confined within the cavity. By this technique, the theoretical, unloaded Q-factor is increased from few thousands up to around $10^{7}$ [50]. Importantly, the mode volume $V_{\mathrm{eff}}\left(r_{\max}\right)$ is almost unchanged by this small displacement of specific holes and remains on the order of the cubic guided wavelength in the case of an L3 cavity [19,50]. The experimental values of the Q-factor are usually lower (owing to fabrication imperfections and impurities [12,50]), but well confirm the optimization trend.

By proper design, PhC cavities with a relatively high Q-factor can be obtained by removing even a single hole (H1 cavity) or no hole at all (H0 cavity) [51]. In particular, an H0 cavity is carved out by slightly shifting two holes in a given row into opposite directions. In addition, the Q-factor of H1 and H0 cavities is optimized by tuning position and radius of the holes surrounding the point-defect. Among PhC cavities, H0 cavities offer the smallest mode volume ($V_{\mathrm{eff}}\left(r_{\max}\right)\approx 0.29{\left(\lambda /n\right)}^{3}$), while keeping a relatively high, unloaded Q-factor ($Q_{i}\approx 10^{5}$) [51].

**Figure 3:**
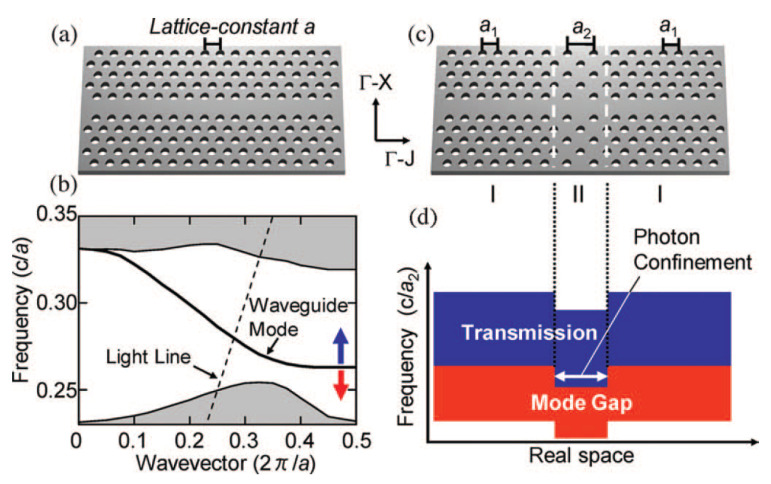
(**a**) Line-defect waveguide and (**b**) corresponding band structure. The blue (red) arrow in (**b**) denotes the pass-band (stop-band), where propagation of photons through the waveguide is allowed (inhibited). (**c**) Photonic heterostructure cavity. (**d**) Schematic representation of the band edges of the photonic heterostructure cavity along the waveguide direction. Reprinted from [12], with the permission of ©IEEE, 2021.

In terms of Q-factor, better performances can be achieved by photonic heterostructure cavities. A so-called *photonic* heterostructure is a connection in series of two or more PhC waveguides with different *photonic* band gaps. The photonic band gap can be engineered by structural changes, such as tuning the lattice constant [52,53], waveguide width [37] or slab refractive index [54]. The working principle is summarized by Figure 3. In this case, the cavity is formed by connecting line-defect waveguides with different lattice constants. Essentially, the larger lattice constant in region II as compared to regions denoted by I (see Figure 3c) induces a relative shift of the band edges corresponding to the various regions (see Figure 3b,d). This ensures an effective confinement of photons along the waveguide direction. In fact, photons which are allowed to propagate in region II find themselves within the band gap of regions I.

Various modifications and improvements of this cavity have been demonstrated. For instance, the lattice constant can be varied more smoothly, by increasing the number of regions which make up the heterostructure. This approach has led to a theoretical, unloaded Q-factor with a record value of $10^{9}$, while keeping a mode volume $V_{\mathrm{eff}}\left(r_{\max}\right)$ around one cubic guided wavelength [55]. More recently, an experimental, unloaded Q-factor with a record value of $1.1\times 10^{7}$ has been even demonstrated [56]. Heterostructure cavities may be also induced via *dynamic* refractive index modulation. This can be done, for instance, by shining an optical pulse at a given location along a line-defect waveguide. Through optical nonlinearities, the refractive index is locally changed and a high-Q heterostructure cavity is formed, which effectively traps photons injected from the waveguide input. With this scheme, a theoretical, unloaded Q-factor even larger than $10^{9}$ has been envisioned [57].

Other types of PhC cavities are nanobeam cavities, as shown in Figure 1b. By perforating a ridge waveguide with a single row of holes, a PhC nanobeam is created [1]. This is a periodic dielectric waveguide, whose guided modes are confined in the lateral and vertical direction by total internal reflection. A cavity is carved out of a PhC nanobeam by introducing a defect. This may consist in one or more missing holes [58], modulation of the hole radius [7,59] or tuning of the lattice constant [60] along the longitudinal direction. As compared to PhC cavities based on line-defect waveguides, PhC nanobeam cavities offer a reduced footprint. EDC cavities may also be obtained out of PhC nanobeam cavities, as shown in Section 6.

**Figure 4:**
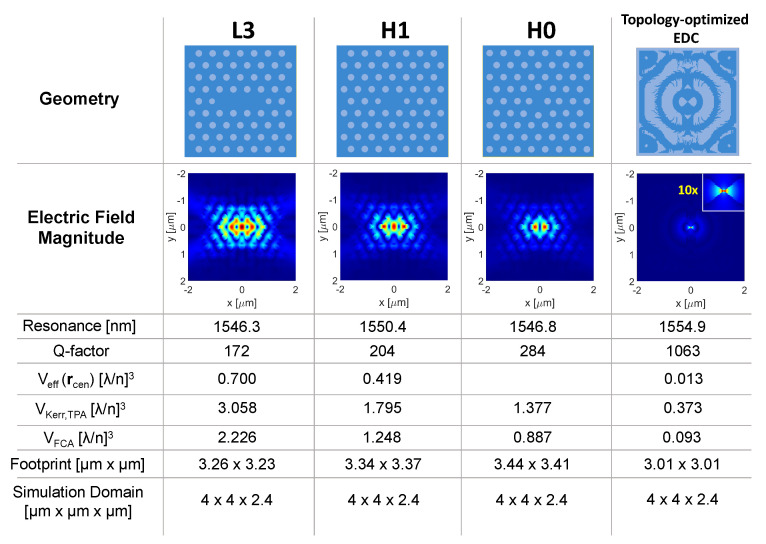
Comparison of three photonic crystal cavities (respectively, L3, H1 and H0) and a cavity for extreme dielectric confinement (EDC) designed by topology optimization. All cavities are made of InP and have similar footprint. We notice, though, that the Q-factors for L3, H1 and H0 may be further optimized by perturbing the holes, as explained earlier. The topology-optimized design was based on that reported in [10], which however has confinement in air. As compared to [10], we added in the center a 10nm×16nm InP bridge. Calculations have been performed by a three-dimensional finite-difference-time-domain (FDTD) method. The center of the cavity is denoted by $r_{\mathrm{cen}}$. The effective mode volume of the H0 cavity is not reported, since the field had a minimum in the center.

Figure 4 compares three PhC cavities (respectively, L3, H1 and H0) against an EDC cavity designed by topology optimization. This structure is very similar to that reported in [10], the difference only being the dielectric bridge in the center, as detailed in the figure caption. We have extracted Q-factor and mode volumes by three-dimensional finite-difference-time-domain (FDTD) calculations, with all cavities made of InP. For a fair comparison, the PhC cavities have been tailored to a similar footprint as the EDC cavity, which resulted in the Q-factor of the PhC cavities being strongly limited by in-plane radiation loss. This comparison displays, though, an original perspective, and points out that EDC cavities may potentially offer a better Q-factor than other PhC cavities of the same size. As for the mode volumes (see Equations (1) and (3)), the simulation domain coincides with the integration volume, *V*; and the volume of the nonlinear material, $V_{\mathrm{nl}}$, is that of InP alone. All mode volumes are smaller for the EDC cavity, which thereby promises enhanced light–matter interactions for various applications. We notice that EDC cavities impose stringent demands to nanofabrication, but very good progress has recently been reported [61].

## 3. Microcavity Lasers for Energy-Efficient Communications

A very important application of PhC cavities is the realization of a new generation of microcavity lasers, e.g., for on-chip optical interconnects [62,63]. In this section we summarize key laser properties, which are determined by the characteristics of the cavity.

The threshold current of an electrically-pumped semiconductor laser may be approximately expressed as [38]

(5) $$I_{\mathrm{th}}=\frac{1}{\tau }\frac{q}{\eta_{i}}\left(N_{\mathrm{tr}}V_{\mathrm{act}}+\frac{\omega_{s}}{Q}\frac{\tau_{r}}{\beta_{\mathrm{sp}}}\right)$$

Here, we have assumed for simplicity the material gain to be linear with carrier density, with $N_{\mathrm{tr}}$ being the transparency carrier density, *q* the electron charge, $\eta_{i}$ the injection efficiency and $\omega_{s}$ the lasing angular frequency. The carrier lifetime, τ, is generally a function of carrier density and related by $\tau^{-1}=\tau_{r}^{-1}+\tau_{nr}^{-1}$ to the radiative lifetime, $\tau_{r}$, and nonradiative lifetime, $\tau_{\mathrm{nr}}$. The spontaneous emission factor, $\beta_{\mathrm{sp}}$, is the fraction of spontaneous emission coupled to the lasing mode. The threshold current has two contributions. The first contribution scales with the active region volume and represents the current required to reach transparency, where stimulated emission and absorption exactly balance. The second contribution is the current necessary to balance the optical loss once transparency has been reached. This term is inversely proportional to the product of Q-factor and spontaneous emission factor. In PhC lasers, $\beta_{\mathrm{sp}}$ is orders of magnitude larger than in conventional semiconductor lasers, with values approaching 1 being reported recently [64,65]. This is because a single dominant mode is found within the bandwidth of spontaneous emission. However, the reduction in threshold current with cavity length is, mostly, simply due to the smaller active volume, which reduces the number of carriers necessary to reach transparency [66]. In fact, the (loaded) Q-factor in PhC lasers is typically high enough ($Q∼10^{3}-10^{4}$ [67]) that the threshold carrier density is essentially determined by the transparency carrier density. It should be noted that the optical confinement factor is embedded implicitly into Equation (5). Indeed, the ratio $\beta_{\mathrm{sp}}/\tau_{r}$ may be equivalently expressed as $\Gamma v_{g}g_{N}/V_{\mathrm{act}}$ [14], with $g_{N}$ being the differential gain [23] and $v_{g}$ the group velocity (reflecting material, but not structural dispersion [14]). Therefore, maximizing the optical confinement factor (thus minimizing the mode volume $V_{p}$) contributes to reducing the threshold current. However, in the presence of a high Q-factor, the improvement is limited and the threshold current is mostly determined by the number of carriers at transparency [28], as already stated.

**Figure 5:**
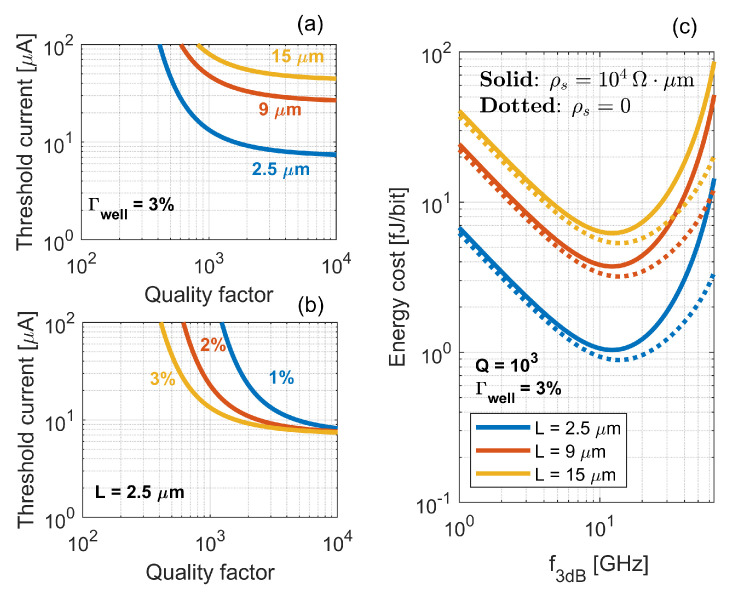
Threshold current versus Q-factor for different values of (**a**) the cavity length, *L*, and (**b**) confinement factor of a single active layer, $\Gamma_{\mathrm{well}}$. In (**a**), $\Gamma_{\mathrm{well}}$ is 3%, and in (**b**) *L* is 2.5μm. (**c**) Energy cost versus 3dB direct modulation bandwidth, with line colors corresponding to different cavity lengths. The electrical resistivity, $\rho_{s}$, is $10^{4}\;\Omega \times \mu m$ (solid) and 0 (dotted). The Q-factor is $Q=10^{3}$ and the confinement factor of a single active layer $\Gamma_{\mathrm{well}}=3\%$.

As an example, we consider the case of a quantum well laser. Figure 5a,b illustrates how the cavity length, *L*, and optical confinement factor per active layer, $\Gamma_{\mathrm{well}}$, impact the threshold current at a given value of the Q-factor. Here, we have assumed a logarithmic dependence of the material gain on carrier density [23]. Furthermore, defect-assisted recombination, radiative recombination and Auger recombination have been modeled, respectively, as linear, quadratic and cubic with carrier density [23]. The simulation parameters closely reflect those of [67] and can be found therein. In particular, we have assumed $N_{\mathrm{well}}=3$, d=6nm, w=300nm and $\alpha_{i}=15\;{\mathrm{cm}}^{-1}$. Here, $N_{\mathrm{well}}$ is the number of active layers, *d* is the thickness of each active layer, *w* is the active region width and $\alpha_{i}$ is the total internal loss. Figure 5a,b emphasizes that increasing the Q-factor or optical confinement factor marginally improves the threshold current if the Q-factor is already high enough. On the contrary, the threshold current is significantly reduced by shortening the cavity length. Overall, PhC lasers have a small threshold current [54,59,60] because they allow one to scale down the active volume, while keeping high the Q-factor and optical confinement factor. It should be noted that heterogeneous [60,63] and epitaxial [6] integration of PhC lasers on silicon have also been demonstrated.

The energy cost, EC, is a common figure of merit encompassing the static and dynamic characteristics of semiconductor lasers in terms of energy efficiency. It is defined as $\mathrm{EC}=P_{\mathrm{in}}/B=\left(IV_{b_{0}}+R_{s}I^{2}\right)/B$ [67]. Here, $B=1.3f_{3\mathrm{dB}}$ is the data rate, which is assumed to be achievable under direct non-return-to-zero modulation [68]. $f_{3\mathrm{dB}}$ is the 3 dB modulation frequency and $P_{\mathrm{in}}=IV_{b_{0}}+R_{s}I^{2}$ is the electrical power. $V_{b_{0}}$ is the built-in bias voltage and $R_{s}$ is the series electrical resistance. This is related to the electrical resistivity, $\rho_{s}$, by $R_{s}=\rho_{s}/L$. Figure 5c illustrates the energy cost as a function of the modulation frequency for $Q=10^{3}$ and $\Gamma_{\mathrm{well}}=3\%$. Each color denotes a different value of the cavity length, with the solid (dotted) line corresponding to $\rho_{s}=10^{4}\;\Omega \times \mu m$ [67] ($\rho_{s}=0$). For simplicity, we have neglected the spontaneous emission coupled to the lasing mode, similarly to [67]. The figure highlights that an optimum modulation bandwidth exists which minimizes the energy cost, as also noted in [62]. An optimum occurs irrespective of $R_{s}$, although a smaller electrical resistance obviously reduces the energy cost. The trend can be easily understood by assuming a low damping factor, leading to $f_{3\mathrm{dB}}\approx \frac{\omega_{R}}{2\pi }\sqrt{1+\sqrt{2}}$ [23], with $\omega_{R}$ being the relaxation resonance frequency. In this case, by neglecting for simplicity the electrical resistance, one may express the energy cost as

(6) $$\mathrm{EC}\approx \left(\frac{I_{\mathrm{th}}}{B}+\frac{B}{\sigma }\right)V_{b_{0}}$$

with $\sigma =\frac{1.69}{4\pi^{2}}\left(1+\sqrt{2}\right)\frac{\eta_{i}\Gamma_{\mathrm{well}}N_{\mathrm{well}}}{qV_{\mathrm{act}}}v_{g}g_{N}$ being a constant independent of the bias current. This equation reveals that at low data rates the power consumption is dominated by the threshold current, with the energy cost being inversely proportional to the data rate. However, as larger data rates are demanded, the current required in excess to threshold increases and gradually dominates the power consumption. Therefore, the energy cost increases as the data rate grows. Overall, Figure 5c emphasizes that an intimate relationship exists among size, speed and energy efficiency [62]. Compact lasers are necessary to minimize the energy cost. In fact, the energy cost benefits twofold from a shorter cavity length, i.e., a smaller active volume. Firstly, via reduction of the threshold current. Secondly, via the enhanced modulation bandwidth at a given current in excess to threshold (i.e., a larger σ). The low threshold current and small active volume equip PhC lasers with reduced energy cost, around 1–10fJ/bit [54,63]. This makes PhC lasers promising light sources for chip-scale optical interconnects [69].

In practice, though, the impact of quantum noise on the laser performance should be also considered [14,70]. For reliable encoding of information, microcavity lasers should be operated well above threshold [71], with a potential increase in the energy cost. The required bit-error rate (BER), depending on the specific application, may also restrain the achievable data rate and further degrade the energy cost [72].

## 4. Line-Defect Cavities

PhC cavities can be analyzed by rigorous approaches, such as FDTD simulations or various frequency domain methods, which directly solve Maxwell’s equations over the entire structure. However, these approaches are computationally demanding. Furthermore, they rely on a global analysis of the cavity, from which gaining physical insights is not always straightforward. On the other hand, it has been shown that the optical confinement in passive LN cavities can be largely understood in terms of a Fabry–Perot picture [73]. Essentially, these cavities behave as effective Fabry–Perot resonators for the *Bloch* modes of the underlying line-defect waveguide. On this basis, we present a transparent and efficient modeling framework to describe the modal properties of passive LN cavities.

### 4.1. Dispersion Relation

**Figure 6:**
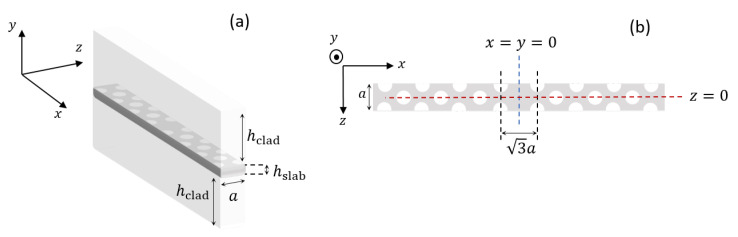
(**a**) Three-dimensional and (**b**) top view of the supercell to compute dispersion relation and Bloch modes of the line-defect waveguide.

Strictly speaking, Bloch’s theorem [2] applies to perfectly periodic lattices. For two-dimensional lattices, this implies that one should assume the crystal to be translationally invariant along the third direction. However, this limitation may be circumvented by the so-called *supercell* method [74]. This method allows one to compute the band structure of PhC slabs and waveguides. Figure 6 shows (a) three-dimensional and (b) top view of the supercell which we have employed. Within the supercell approximation, the supercell is periodically replicated in all the three dimensions and the band structure of this artificial three-dimensional lattice is computed. The supercell consists of a single lattice constant *a* along *z* direction, along which the waveguide is indeed periodic. The dimensions along *x* and *y* directions should instead be chosen so as to ensure a significant decay of the guided modes towards the boundaries. This condition can be easily met, since the guided modes are strongly localized to the line-defect.

With these choices, the eigenmodes of the artificial three-dimensional lattice well approximate those of the line-defect waveguide. We assume the simulation parameters in Table 1, reflecting the PhC lasers of [75]. Figure 2a shows the band structure for TE-like modes, computed by the plane wave-expansion method [43]. In the following, we focus on the lower-frequency guided mode, denoted by red in Figure 2a.

### 4.2. Bloch Modes

The plane wave expansion method provides the Bloch modes in a fully vectorial form (that is, *x*, *y* and *z* components) and with three-dimensional spatial dependence, namely,

(7) $$E_{\pm }\left(r\right)=e_{\pm }\left(r\right)e^{\pm ik_{z}z}$$

Here, $E_{+}$ ($E_{-}$) is the electric field of the forward-propagating (backward-propagating) guided mode. The envelopes $e_{\pm }$ are *z* periodic, with the period given by the lattice constant, *a*. $k_{z}$ is the wavenumber along the *z* axis. The Bloch modes are normalized, at each frequency, such that $\int_{V_{\mathrm{cell}}}\epsilon_{0}n_{b}^{2}\left(r\right){\left|e_{+}\left(r\right)\right|}^{2}dV_{\mathrm{cell}}=1$. Here, $V_{\mathrm{cell}}$ is the volume of the supercell and $n_{b}$ the background refractive index, with $n_{b}=n_{\mathrm{slab}}$ ($n_{b}=n_{\mathrm{clad}}$) in the slab (cladding). For a qualitative understanding of the radiation loss in LN cavities, it suffices to consider the lateral field component along the longitudinal direction [19,47]. Therefore, in the following we drop the vectorial notation and implicitly refer to the lateral field component along the *centerline* of the waveguide. This line is denoted by x=y=0 in Figure 6b, with y=0 corresponding to the middle of the slab along the vertical direction.

**Figure 7:**
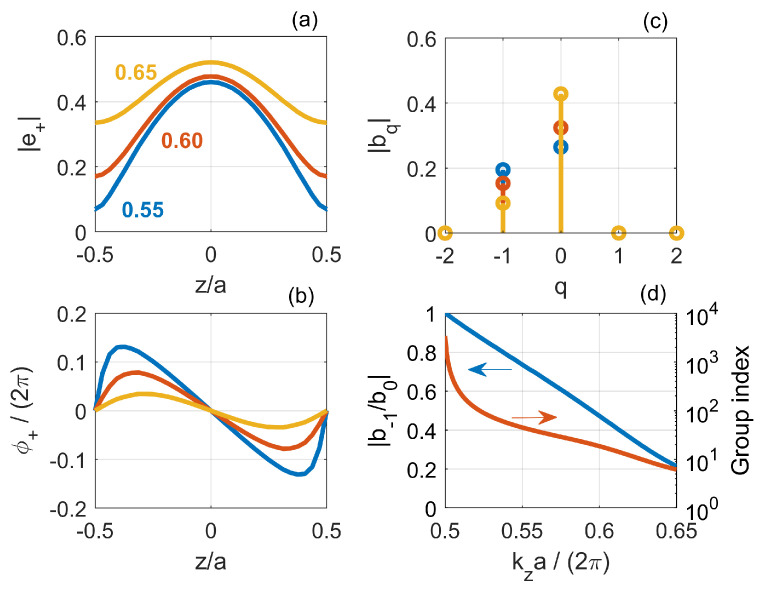
Bloch modes of the line-defect waveguide. (**a**) Magnitude and (**b**) phase of the lateral electric field component of the forward-propagating Bloch mode along the centerline of the waveguide. Each color corresponds to a different value of the wavenumber, which is reported in (**a**) in units of 2π/a. (**c**) Magnitudes of the spatial Fourier harmonics $b_{q}$. (**d**) Magnitudes of the ratio between the spatial harmonic of order −1 and the average (left). Group index (right).

Figure 7 shows (a) the magnitude and (b) phase of the forward-propagating Bloch mode in a unit cell as a function of *z*. Here, z=0 is the center of the supercell along the longitudinal direction, as indicated in Figure 6b. Each color corresponds to a different value of the wavenumber, reported in units of 2π/a. These figures highlight that, as the wavenumber approaches the band edge at π/a, (1) the peak-to-peak amplitude increases and (2) the phase linearly varies with *z* over a larger and larger portion of the unit cell. These features are common to Bloch modes of conventional Bragg gratings [76]. Moreover, whichever the frequency, the phase is zero at the input and output of the unit cell. Being periodic, the Bloch modes can be expanded in a Fourier series, namely $e_{+}\left(z\right)=\sum_{q}b_{q}e^{+iq\frac{2\pi }{a}z}$. The spatial harmonics, $b_{q}$, are reported in Figure 7c. The harmonics with *q* other than −1 and 0 are negligible. Therefore, we may express $e_{+}\left(z\right)$ as

(8) $$e_{+}\left(z\right)=b_{0}+b_{-1}e^{-i\frac{2\pi }{a}z}$$

Furthermore, as a result of the variation in the peak-to-peak amplitude (see Figure 7a) and the linear z−dependence of the phase (see Figure 7b), the magnitude of $b_{-1}$ approaches the spatial average, $b_{0}$, as the wavenumber moves towards the band edge. The physical interpretation of this behavior is straightforward. Upon insertion of Equation (8) into Equation (7), the forward-propagating Bloch mode reads

(9) $$E_{+}\left(z\right)=\underset{\mathrm{FW}}{\underset{︸}{b_{0}e^{+ik_{z}z}}}+\underset{\mathrm{BW}}{\underset{︸}{b_{-1}e^{-i\left(\frac{2\pi }{a}-k_{z}\right)z}}}$$

In other terms, a Bloch mode results from the interference of a backward (BW) and forward (FW) component [77], whose *relative strength* corresponds to the ratio $b_{-1}/b_{0}$. If the mode as a whole is forward-propagating, then the forward component is stronger, i.e., $|b_{-1}/b_{0}|<1$. In the limit $|b_{-1}/b_{0}|=1$, the backward and forward component balance out. Thus, the *net* velocity at which the mode as a whole propagates, which is the group velocity, is zero and the mode is a standing wave. This is the reason why $|b_{-1}/b_{0}|$ tends to 1 as $k_{z}$ approaches the band edge, as shown (left axis) by Figure 7d. The ratio $b_{-1}/b_{0}$ encodes the composition of the Bloch mode, which depends on the waveguide geometry and detuning from the band edge. Figure 7d also displays (right axis) the group index, which for a perfect structure, without any disorder, diverges at the band edge.

### 4.3. Resonance Condition

**Figure 8:**
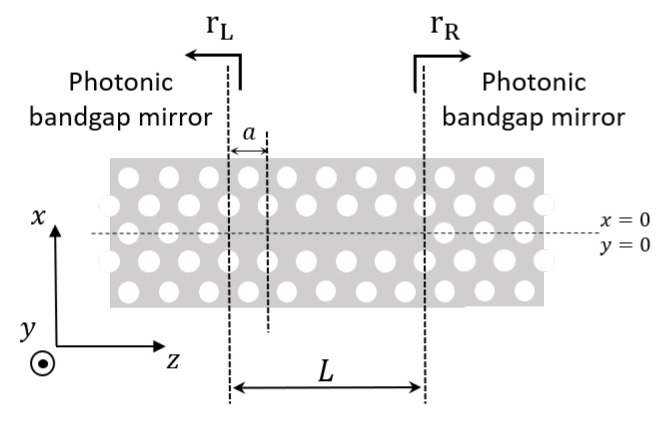
LN cavity, with N denoting the number of missing holes. The figure illustrates our choice of the reference planes for the cavity length *L*.

Figure 8 shows a top view of an LN cavity. *L* is the cavity length, and $r_{L}$ ($r_{R}$) is the left (right) mirror’s reflection coefficient. These mirrors are broadband and generally have a high reflectivity [73]. We expand the field in the basis of the two guided counter-propagating Bloch modes. In the absence of optical gain and disorder, the Bloch modes are only coupled at the mirrors. In this case, one finds

(10) $$E\left(z\right)=\tilde{F}e^{+ik_{z}z}e_{+}\left(z\right)+\tilde{B}e^{-ik_{z}z}e_{-}\left(z\right)$$

where while $\tilde{F}$ ($\tilde{B}$) is the amplitude of the forward (backward) Bloch mode. We set the point z=0 at the center of the cavity. Therefore, the left (right) mirror is located at z=−L/2 (z=L/2) and the boundary conditions read

(11a) $$\begin{array}{cc} \; & \tilde{F}e^{+ik_{z}L/2}e_{+}\left(L/2\right)r_{R}=\tilde{B}e^{-ik_{z}L/2}e_{-}\left(L/2\right) \end{array}$$

(11b) $$\begin{array}{cc} \; & \tilde{B}e^{-ik_{z}\left(-L/2\right)}e_{-}\left(-L/2\right)r_{L}=\tilde{F}e^{+ik_{z}\left(-L/2\right)}e_{+}\left(-L/2\right) \end{array}$$

The backward mode, $e_{-}\left(z\right)$, is the complex conjugate of $e_{+}\left(z\right)$ [78]. By using this property and requiring that the equations have a solution other than the trivial solution $\tilde{F}=\tilde{B}=0$, one finds

(12) $$r_{L}r_{R}e^{+2i\left[\phi_{+}\left(\frac{L}{2}\right)-\phi_{+}\left(-\frac{L}{2}\right)\right]}e^{+2ik_{z}L}=1$$

where $\phi_{+}\left(z\right)$ is the phase of $e_{+}\left(z\right)$. With our choice of a unit cell, $\phi_{+}\left(z\right)$ is zero at the unit cell input and output (see Figure 7b). Therefore, one finds $\phi_{+}\left(\pm L/2\right)=0$. Since *L* consists of an integer number of unit cells (see Figure 8) and if the phase of the left and right mirror is equal to either zero or π, from Equation (12) one obtains

(13) $$k_{z}-\pi /a=m\pi /L$$

in agreement with [47,75]. Here, *m* is an integer. Equation (13) provides the detuning of the wavenumber $k_{z}$ from the band edge for the longitudinal resonant mode of order *m*. As the cavity length increases, this detuning diminishes. Correspondingly, the frequency of the resonant mode decreases, by following the dispersion relation of the underlying line-defect waveguide (see Figure 2a, red line). Importantly, FDTD simulations of LN cavities have revealed that the resonant modes do obey Equation (13) to good approximation [47]. Therefore, we take this agreement as an indication that the phase of the left and right mirror reflection coefficient in Figure 8 may be approximated with either zero or π. In Section 4.4, we show that zero is the right approximation.

### 4.4. Resonant Modes: Real Space Distribution

In the following, we derive the spatial dependence of the electric field of a resonant mode. First of all, we assume that an integer number of periods of the Bloch modes $e_{\pm }\left(z\right)$ fits into the cavity length, *L*, as denoted in Figure 8. Secondly, we assume the mirrors to have (1) frequency-independent reflection coefficients and (2) zero penetration length. In practice, the field is evanescent within the mirrors, with a frequency-dependent decay constant [53]. However, here we are only interested in the field spatial dependence within the cavity length, which is enough to capture the essential physics.

Under these simplifying assumptions and by usage of Equation (10), the electric field within the cavity may be expressed as

(14) $$E\left(z\right)=w\left(z,L\right)\left[\tilde{F}e^{+ik_{z}z}e_{+}\left(z\right)+\tilde{B}e^{-ik_{z}z}e_{-}\left(z\right)\right]$$

Here, the window function w(z,L) accounts for the field confinement, with w=1 (w=0) for |z|≤L/2 (|z|>L/2). We denote the wavenumber $k_{z}$ of a resonant mode as $k_{2}$ and insert Equation (8) into Equation (14), leading to

(15) $$E\left(z\right)=w\left(z,L\right)\left[\tilde{F}\left(b_{0}e^{+ik_{2}z}+b_{-1}e^{-i\frac{2\pi }{a}z}e^{+ik_{2}z}\right)+\tilde{B}\left(b_{0}^{*}e^{-ik_{2}z}+b_{-1}^{*}e^{+i\frac{2\pi }{a}z}e^{-ik_{2}z}\right)\right]$$

where * denotes the complex conjugate. By using $\phi_{+}\left(\pm L/2\right)=0$, the boundary condition at the right mirror from Equation (11a) can be recast as $\tilde{B}=\tilde{F}e^{+ik_{2}L}r_{R}$. By inserting this expression into Equation (15), the electric field of a resonant mode reads

(16) $$E\left(z\right)=\tilde{F}w\left(z,L\right)\left[\left(b_{0}e^{+ik_{2}z}+b_{-1}e^{-ik_{1}z}\right)+\left(b_{0}^{*}e^{-ik_{2}z}+b_{-1}^{*}e^{+ik_{1}z}\right)r_{R}e^{+ik_{2}L}\right]$$

where we have defined the wavenumber

(17) $$k_{1}=2\pi /a-k_{2}$$

In addition, since L=Na, the resonance condition from Equation (13) can be expressed as $k_{2}L=\left(m+N\right)\pi$ with *N* being the number of unit cells. This condition, together with Equations (16) and (17), provide the electric field distribution of the *m*-th longitudinal mode in an LN cavity. For the sake of brevity, in the following we focus on the fundamental mode, i.e., m=1, but the same analysis can be easily applied to higher-order modes.

We assume the magnitude of the right mirror reflection coefficient $r_{R}$ to be equal to 1, which is a reasonable approximation for photonic band gap mirrors [73]. Concerning the phase, we take it to be equal to zero. We discuss later the implications of a phase equal to π. Since m=1, one finds $e^{+ik_{2}L}$ to be equal to 1 (−1) if *N* is odd (even). Moreover, if *N* is odd (even), the center of the cavity at z=0 is aligned with a maximum (minimum) in the magnitude of the Bloch mode $e_{+}\left(z\right)$. Thus, one finds the spatial harmonic $b_{-1}$ to be real and positive (real and negative) if *N* is odd (even). The spatial average $b_{0}$ is real and positive irrespective of *N* being either odd or even. In light of these considerations, from Equation (16) the fundamental resonant mode reads

(18) $$E\left(z\right)=\left\{\begin{array}{cc} 2\tilde{F}w\left(z,L\right)\left[|b_{0}|\cos\left(k_{2}z\right)+\left|b_{-1}\right|\cos\left(k_{1}z\right)\right], & \mathrm{for}\;N\;\mathrm{odd}\\ 2i\tilde{F}w\left(z,L\right)\left[|b_{0}|\sin\left(k_{2}z\right)+\left|b_{-1}\right|\sin\left(k_{1}z\right)\right], & \mathrm{for}\;N\;\mathrm{even} \end{array}\right.$$

**Figure 9:**
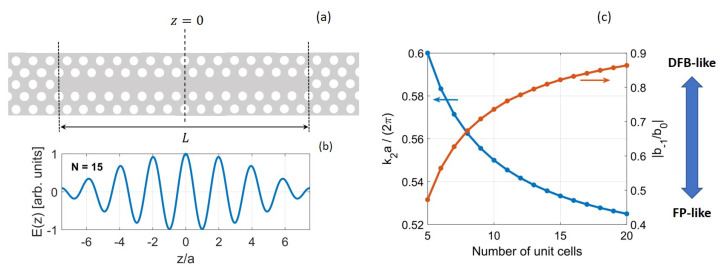
(**a**) L15 cavity and (**b**) electric field profile of the fundamental resonant mode. (**c**) Resonance condition of LN cavities: normalized wavevector $k_{2}$ (**left**) and magnitude of the ratio between the Bloch modes spatial harmonic $b_{-1}$ and spatial average $b_{0}$ (**right**).

For a given *N*, the fundamental mode has the smallest detuning from the band edge and thus the lowest frequency. For *N* being odd (even), the mode is even (odd) with respect to the center of the cavity, as also found in [47]. Figure 9a,b shows an L15 cavity and the corresponding electric field profile for the fundamental resonant mode.

The field pattern results from the interference of two pairs of plane waves, with wavevectors $\pm k_{1}$ and $\pm k_{2}$. The amplitude of the plane waves with wavevector $\pm k_{2}$ ($\pm k_{1}$) is the magnitude of the spatial average $b_{0}$ (the spatial harmonic $b_{-1}$). Therefore, the ratio $|b_{-1}/b_{0}|$ represents the relative strength of one pair of plane waves as compared to the other. As the number of unit cells is varied, the resonance frequency changes and the ratio $|b_{-1}/b_{0}|$ adjusts itself accordingly. This is emphasized by Figure 9c, which displays $k_{2}$ (left) and $|b_{-1}/b_{0}|$ (right) versus the number of unit cells.

From Equations (13) and (17), the wavevectors $k_{1}$ and $k_{2}$ read $k_{1}=\pi /a-\pi /L$ and $k_{2}=\pi /a+\pi /L$, respectively. As *L* increases, both $k_{1}$ and $k_{2}$ move towards the band edge. Correspondingly, $|b_{-1}|$ tends to $|b_{0}|$ (see Figure 7d) and the fundamental mode gradually turns distributed-feedback-like (DFB-like). This attribution [47] stems from the fact that the mode distribution in a DFB laser is determined by *two* pairs of wavevectors [23]. On the contrary, the shorter the cavity is, the more a single pair of plane waves (that with wavevectors $\pm k_{2}$) dominates over the other. Therefore, the fundamental mode turns Fabry–Perot-like. Indeed, in a Fabry–Perot laser the mode distribution is determined by a single pair of plane waves. Resonance condition (see Equation (13)) and electric field distribution (see Equation (18)) are consistent with previous FDTD simulations [47]. In particular, the ratio $|b_{-1}/b_{0}|$ (see Figure 9c) is obtained in [47] by fitting. Here, we have elucidated the physical origin of this parameter, which directly stems from the spatial and frequency dependence of the Bloch modes of the underlying line-defect waveguide.

As mentioned at the end of Section 4.3, the phase of the mirrors at their respective reference planes in Figure 8 can be considered to be either zero or π to good approximation. In this section, we have assumed the phase of the right mirror to be zero. Assuming this phase to be π would invert the parity of the mode with respect to the center of the cavity. In this case, the parity would be inconsistent with the FDTD simulations of [47]. We view this result as a hint that the phase of the right and left mirror, as defined by the interfaces in Figure 8, is indeed zero to good approximation.

### 4.5. Resonant Modes: Reciprocal Space Distribution and Radiation Loss

**Figure 10:**
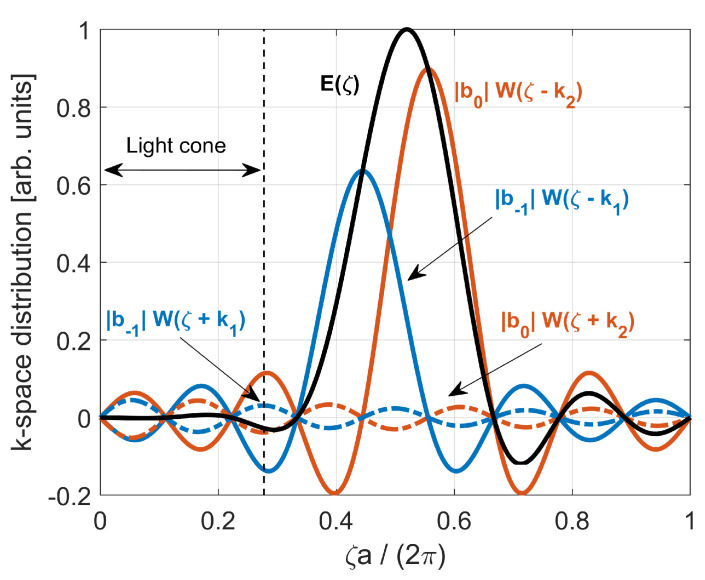
Components of the *k*-space distribution for an L9 cavity computed by Equation (19). The blue (red) solid line is for the sinc function centered at $+k_{1}$ ($+k_{2}$). The blue (red) dotted line corresponds to the sinc function centered at $-k_{1}$ ($-k_{2}$). The black solid line is the total spectrum. The black dashed line delimits the light cone.

The reciprocal or *k*-space distribution is the Fourier transform of the spatial field. From Equation (18), the Fourier transform of E(z) reads

(19) $$\begin{array}{cc} \; & \begin{array}{cc} E\left(\zeta \right)=\tilde{F} & \left[|b_{0}|W\left(\zeta -k_{2}\right)+\left|b_{0}\right|W\left(\zeta +k_{2}\right)\right.\\ \; & +\left.|b_{-1}|W\left(\zeta -k_{1}\right)+\left|b_{-1}\right|W\left(\zeta +k_{1}\right)\right],\;\mathrm{for}\;N\;\mathrm{odd} \end{array} \end{array}$$

(20) $$\begin{array}{cc} \; & \begin{array}{cc} E\left(\zeta \right)=\tilde{F} & \left[|b_{0}|W\left(\zeta -k_{2}\right)-\left|b_{0}\right|W\left(\zeta +k_{2}\right)\right.\\ \; & +\left.|b_{-1}|W\left(\zeta -k_{1}\right)-\left|b_{-1}\right|W\left(\zeta +k_{1}\right)\right],\;\mathrm{for}\;N\;\mathrm{even} \end{array} \end{array}$$

where ζ is the spatial angular frequency and $W\left(\zeta \right)=L\frac{\sin\left(\zeta L/2\right)}{\zeta L/2}=L\mathrm{sinc}\left(\frac{\zeta L}{2\pi }\right)$ the spatial Fourier transform of w(z,L). For a given cavity length, the light cone is given by $|\zeta |<\left(\omega_{0}/c\right)n_{\mathrm{clad}}$, with $\omega_{0}$ being the angular frequency of the resonant mode. As an example, we select an L9 cavity and plot in Figure 10 the various components of the mode spectrum from Equation (19). The blue (red) solid line is for the sinc function centered at $+k_{1}$ ($+k_{2}$), and the blue (red) dotted line corresponds to that centered at $-k_{1}$ ($-k_{2}$). The total spectrum is the black line. Only the positive frequencies are shown due to the symmetry of the spectrum. We note that the destructive interference of the various components contributes to reducing the field spectral content within the light cone.

We define the *light cone power fraction* as the relative fraction of the electric field intensity within the light cone, namely $\eta =\int_{-\zeta_{0}}^{+\zeta_{0}}{\left|E\left(\zeta \right)\right|}^{2}d\zeta /\int_{-\infty }^{+\infty }{\left|E\left(\zeta \right)\right|}^{2}d\zeta$, where $\zeta_{0}=\left(\omega_{0}/c\right)n_{\mathrm{clad}}$ is the upper limit of the light cone for a given cavity length. By computing the light cone power fraction as a function of the cavity length, one obtains an approximate measure of the scaling of the radiation loss with the size of the cavity [75]. The absolute radiation loss rate and, therefore, the Q-factor, may be quantified through full 3D-simulations of the structure (see, e.g., [79] for a comparison of different numerical methods and their accuracies), but this falls outside the scope of this paper.

**Figure 11:**
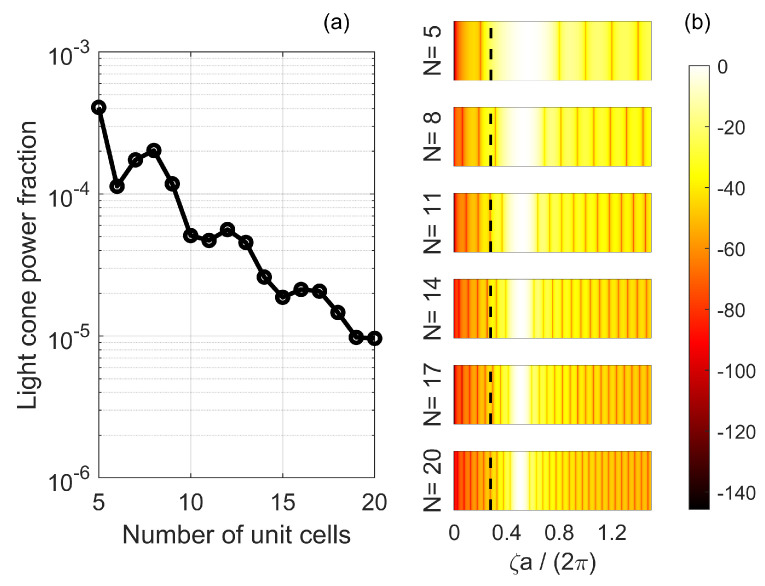
(**a**) Light cone power fraction as a function of the size of the cavity. (**b**) Squared magnitude of the electric field *k*-space distribution in dB normalized to its maximum for various LN cavities. The dashed, vertical line indicates the upper limit of the light cone for each cavity.

The result is illustrated in Figure 11a. The light cone power fraction decreases as the size of the cavity increases, albeit with some local maxima. Depending on the ratio $|b_{-1}/b_{0}|$, the various components of the field *k*-space distribution (shown in Figure 10 for an L9 cavity) interfere *destructively* within the light cone, more or less effectively. This mechanism is the origin of the local maxima in Figure 11a, which were also found in [75]. The general reduction of the light cone power fraction with increasing cavity length is due to the *k*-space distribution being shifted outside the light cone. This is emphasized by Figure 11b, depicting ${\left|E\left(\zeta \right)\right|}^{2}$ in dB normalized to its maximum value for various LN cavities. The spectrum is computed through Equations (19) and (20). The dashed, vertical line denotes the upper limit of the light cone for each cavity. As the cavity becomes longer, (1) $k_{1}$ approaches π/a and (2) the width of each of the sinc functions in Equations (19) and (20) is reduced. As a result, the field spectrum departs from the light cone and the light cone power fraction generally decreases.

We note that FDTD simulations have demonstrated monotonic increase in Q-factor with cavity length, without local maxima [47,79]. This is because, in practice, the destructive interference of the various spatial frequency components of the field is less effective than predicted by Equations (19) and (20). As a result, the Q-factor is essentially determined by the shift of the field *k*-space distribution outside the light cone.

### 4.6. Slow-Light Photonic Crystal Lasers

In Section 3, we have outlined the main advantages of microcavity lasers, such as PhC lasers. Slow-light is a further, potential advantage of PhC lasers. In fact, the gain coefficient per unit length is enhanced when operating in a regime of slow-light, as originally pointed out in [80]. This effect has been verified experimentally in active PhC waveguides [81]. Intuitively, the gain enhancement results from a longer effective length, due to the multiple back-and-forth scattering across the periodic structure. As a result, PhC lasers [75] and on-chip optical amplifiers [82] may benefit from slow-light in terms of compactness and energy efficiency. Notice that light slow-down in translationally invariant waveguides, e.g., close to a mode cut-off, does not enhance the spatial gain [83].

A critical limitation to the use of slow-light is the presence of disorder [44], i.e., fabrication imperfections which inevitably affect real devices. These imperfections can be, for instance, roughness at the hole boundary or displacement of holes from the position of the ideal periodic lattice, on the order of few nanometers. Disorder may induce two types of loss [84], namely, out-of-plane radiation loss and backscattering loss. In the latter case [85,86], a guided mode propagating in a given direction is partly reflected by the waveguide imperfections, thereby losing power due to coupling with guided, counter-propagating modes. Furthermore, an intrinsic limitation to the slow-light gain enhancement is posed by the gain itself [87]. This effect has been analysed in [88] for slow-light semiconductor optical amplifiers. As shown therein, the presence of material gain induces a distributed backscattering, which ultimately limits the amplifier gain.

In the following, we briefly discuss the impact of slow-light on threshold gain and out-coupling efficiency of line-defect PhC lasers. We neglect the influence of the gain-induced distributed feedback, which has been shown to be small, unless the lasing frequency is very close to the band edge [89]. Furthermore, we also neglect the disorder-induced backscattering loss, the impact of which is currently a matter of debate [75,90]. Proper inclusion of this effect is possible by combining descriptions of gain-induced [88] and disorder-induced [85] distributed feedback, which is beyond the scope of this paper.

**Figure 12:**
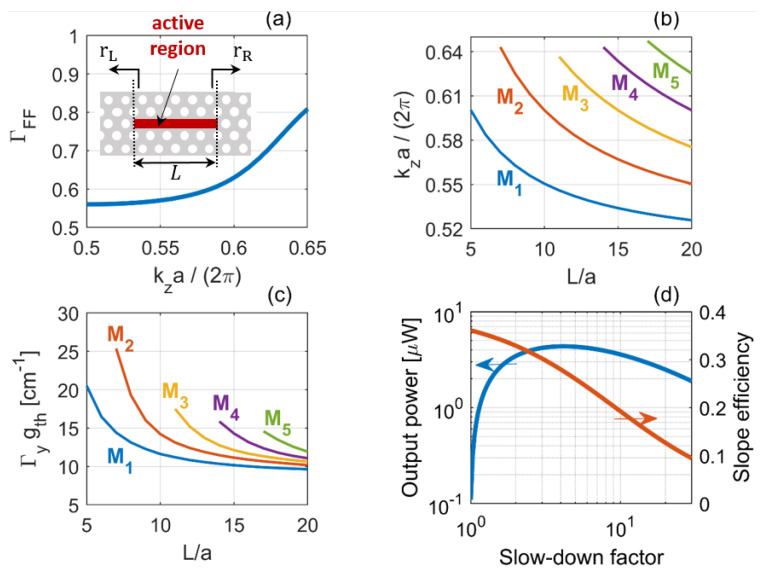
(**a**) Optical confinement factor $\Gamma_{\mathrm{FF}}$ of a line-defect PhC laser with a buried heterostructure active region (inset). $\Gamma_{\mathrm{FF}}$ was computed as if the active layers entirely filled the slab in the vertical (*y*) direction. The *x*-axis shows the wavenumber $k_{z}$ of the associated line-defect waveguide without active material. (**b**) Wavenumber and (**c**) modal gain at the lasing threshold of the various longitudinal modes versus cavity length. The modal gain only included the optical confinement factor $\Gamma_{y}$, which was approximately independent of frequency. $\Gamma_{y}$ accounts for the optical confinement along the vertical direction within the active layers. (**d**) Output power (**left**) and slope efficiency (**right**) versus the slow-down factor. The cavity length was L=5a and the bias current was 50 μA. Further details on the simulation parameters are provided within the text.

We consider line-defect PhC lasers with a buried heterostructure active region, as shown in the inset of Figure 12a. In the absence of gain-induced distributed feedback, the waveguide complex propagation constant reads [88]

(21) $${\tilde{\beta }}_{\mathrm{eff}}\left(\omega ,N_{\mathrm{car}}\right)=k_{z}\left(\omega \right)-\frac{i}{2}S\left(\omega \right)\left[\Gamma_{\mathrm{FF}}\left(\omega \right)\Gamma_{y}g\left(N_{\mathrm{car}}\right)\left(1-i\alpha_{H}\right)-\alpha_{1}\right]$$

Here, $S=n_{g}/n_{\mathrm{slab}}$ is the *slow-down factor*, where $n_{g}$ is the group index of the line-defect waveguide without active material (see Figure 7d, right axis); *g* is the material gain, which depends on the carrier density, $N_{\mathrm{car}}$; $\alpha_{H}$ is the linewidth enhancement factor; and $\alpha_{1}$ is the disorder-induced out-of-plane radiation loss [91]. Optical confinement along the vertical direction within the active layers is accounted for by $\Gamma_{y}$. On the other hand, $\Gamma_{\mathrm{FF}}$ is the optical confinement factor computed *as if* the active layers entirely filled the slab along the vertical direction. Owing to the high refractive index contrast between slab and cladding, $\Gamma_{y}$ is essentially independent of frequency. For instance, for a single active layer of 8nm placed in the middle of the slab, we estimate $\Gamma_{y}\approx 4\%$. On the contrary, $\Gamma_{\mathrm{FF}}$ does depend on frequency and diminishes towards the band edge [88,92], as shown by Figure 12a. This is due to spreading of the Bloch modes in the lateral direction as the frequency approaches the band edge. The laser oscillation condition, i.e., $r_{L}r_{R}e^{+2i{\tilde{\beta }}_{\mathrm{eff}}\left(\omega ,N_{\mathrm{car}}\right)L}=1$, leads to

(22a) $$\begin{array}{cc} \; & \Gamma_{\mathrm{FF}}\Gamma_{y}g_{\mathrm{th}}=\alpha_{1}+\alpha_{\mathrm{mir}}/S \end{array}$$

(22b) $$\begin{array}{cc} \; & 2\left(k_{z}-S\Gamma_{\mathrm{FF}}\Gamma_{y}g_{\mathrm{th}}\alpha_{H}/2-\pi /a\right)L+\phi_{L}+\phi_{R}=2\pi m \end{array}$$

Here, $\alpha_{\mathrm{mir}}=\frac{1}{L}\ln\left(\frac{1}{|r_{L}\left|\right|r_{R}|}\right)$ is the mirror loss in the absence of slow-light, $\phi_{L}$ ($\phi_{R}$) is the phase of the left (right) mirror and the integer *m* is the order of the resonant mode. Equations (22a) and (22b) are the amplitude and phase condition, respectively. All quantities are evaluated at the oscillation frequency. As an example, we have assumed $r_{L}=1$, $r_{R}=0.99$, $\phi_{L}=\phi_{R}=0$, $\alpha_{1}=5\;{\mathrm{cm}}^{-1}$ and $\alpha_{H}=1.5$. The other parameters of the line-defect waveguide are summarized in Table 1. For a given *m*, as the cavity length increases, a smaller detuning from the band edge at π/a is required, in order to continue to fulfill the phase condition. Therefore, the modes move towards the band edge with increasing cavity length, as shown by Figure 12b, where line colors correspond to different modes. This trend is in agreement with experimental results [75]. Correspondingly, the modal threshold gain $\Gamma_{y}g_{\mathrm{th}}$ is reduced, due to the diminishing *effective* mirror loss, $\alpha_{\mathrm{mir}}/S$. This is illustrated by Figure 12c. The effective loss decreases because of the larger cavity length (as for conventional semiconductor lasers) *and* enhanced slow-down factor, due to the shift of the oscillation frequency towards the slow-light region. This benefit outweighs the reduction of the optical confinement factor $\Gamma_{\mathrm{FF}}$.

The slow-light enhancement of the gain per unit length equivalently manifests itself as a reduction of the cavity loss per unit time, i.e., a longer photon lifetime. Specifically, one finds [75,93]

(23) $$\frac{1}{\tau_{p}}=\frac{\omega_{s}}{Q}=\underset{\omega_{s}/Q_{c}}{\underset{︸}{\left(c/n_{\mathrm{slab}}\right)\alpha_{\mathrm{mir}}/S}}+\underset{\omega_{s}/Q_{i}}{\underset{︸}{\left(c/n_{\mathrm{slab}}\right)\alpha_{1}}}$$

Therefore, slow-light increases the Q-factor associated with out-coupling losses, $Q_{c}$. With these definitions, one may utilize conventional rate equation models [23] to compute key laser properties, such as threshold current and output power. In particular, the threshold current reads (cf. Equation (5)) $I_{\mathrm{th}}=\frac{1}{\tau }\frac{q}{\eta_{i}}\left(N_{\mathrm{tr}}V_{\mathrm{act}}+\frac{\omega_{s}}{Q}\frac{V_{\mathrm{act}}}{\Gamma \left(c/n_{\mathrm{slab}}\right)g_{N}}\right)$. Here, Γ is equal to $\Gamma_{\mathrm{FF}}\Gamma_{y}$ and *Q* is given by Equation (23). The output power is expressed as $P_{\mathrm{out}}=\left(\hbar \omega_{s}/q\right)\;\mathrm{SE}\times \left(I-I_{\mathrm{th}}\right)$, where $\mathrm{SE}=\eta_{i}\frac{Q_{i}}{Q_{i}+Q_{c}}$ is the slope efficiency.

To single out the impact of the slow-down on output power and slope efficiency, we select a given cavity length. By tuning the mirror phases, one may vary the oscillation frequency and subsequently the slow-down factor, with the cavity length being fixed. As an example, Figure 12d displays the output power (left) and slope efficiency (right) for L=5a versus the slow-down factor. Here, we have assumed the active region to consist of 3 active layers, each being 8 nm thick, leading to $\Gamma_{y}=12\%$. Furthermore, we have neglected the variation of $\Gamma_{\mathrm{FF}}$ with the oscillation frequency and assumed $\Gamma_{\mathrm{FF}}\approx 63\%$. The active region width is w=500nm. Other parameters are $g_{N}=5\times 10^{24}\;{\mathrm{cm}}^{2}$, $N_{\mathrm{tr}}=10^{18}\;{\mathrm{cm}}^{-3}$, τ=0.5ns and $\eta_{i}=0.4$, and the bias current is I=50μA. The output power initially builds up as the slow-down factor grows, owing to the increasingly smaller threshold current. However, as the slow-down factor is further increased, the output power saturates and subsequently diminishes, because of the degradation of the slope efficiency. Consequently, while a larger slow-down factor always improves the threshold current, an optimum value exists which maximizes the output power.

We notice that experimental results on PhC lasers have shown an optimum cavity length, where the threshold density is minimum [75]. This is interpreted as being due to enhanced backscattering loss as the slow-down factor increases. A dscussion of this effect is outside the scope of this paper.

## 5. Fano Laser

**Figure 13:**
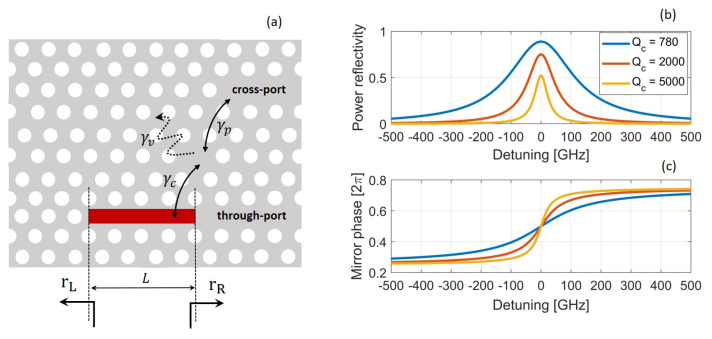
(**a**) Fano laser implementation on the PhC platform. The active region is denoted in red. (**b**) Power reflectivity and (**c**) phase for a Fano mirror with $Q_{v}=10^{5}$, $Q_{p}=1.5\times 10^{4}$ and different values of $Q_{c}$ versus detuning from the resonance frequency.

In this section, we analyze key properties of a new, recently proposed PhC laser, the so-called Fano laser [94]. The essence of this laser is the Fano mirror, realized through coupling of a waveguide with a nanocavity located adjacently. A schematic representation is shown in Figure 13a. The nanocavity is typically realized in the form of an H1 or H0 cavity. However, one might also imagine to use an EDC cavity, so as to enhance the ratio between Q-factor and mode volume. The Fano resonance is a general wave interference phenomenon [95,96]. The Fano mirror reflection does not arise from a discontinuity in refractive indices, but rather from the interference between two light paths: the direct waveguide path and the indirect waveguide-nanocavity-waveguide path. Around the nanocavity resonance frequency, the interference of these two paths is *destructive*. As a result, a narrowband reflection spectrum arises. By implementing the Fano laser on a PhC platform, the rich physics of the Fano mirror is combined with the opportunities offered by the PhC technology. Among the intriguing properties of the Fano laser (still to be fully explored), we cite ultrafast frequency modulation [94,97], increased stability against external optical feedback [98] and passive [99] and active Q-switching [100]. A comprehensive review of the theory and current experimental status can be found in [40]. Recently, the Fano laser has been also experimentally demonstrated to possess ultra-narrow linewidth [101]. Furthermore, we note that the Fano mirror lends itself to various optical signal processing applications, which are thoroughly reviewed in [102].

### 5.1. Fano Mirror

The Fano mirror can be modeled by temporal coupled-mode theory [103]. The most general implementation of the Fano mirror on the PhC platform may include a blocking air hole in the waveguide below the nanocavity (a so-called PTE, partially transmitting element) [104]. This PTE renders the mirror spectrum asymmetric with respect to the nanocavity resonance frequency, which can be useful in various applications [100,102]. By displacing the PTE from the mirror plane, the parity of the resonance can also be controlled, thereby blue or red-shifting the reflection maximum as compared to the minimum.

In the following, we focus on the simplest implementation, without a PTE. In this case, one finds [94]

(24) $$r_{R}\left(\omega \right)=\frac{-\gamma_{c}}{i\left(\omega_{0}-\omega \right)+\gamma_{T}}$$

Here, $\omega_{0}$ is the nanocavity resonance frequency, $\gamma_{c}$ is the coupling rate between nanocavity and waveguide, and $\gamma_{T}=\gamma_{c}+\gamma_{v}+\gamma_{p}$ is the total decay rate of the field in the nanocavity. This rate accounts for coupling with the waveguide ($\gamma_{c}$), vertical out-of-plane scattering loss ($\gamma_{v}$) and possible coupling to other ports ($\gamma_{p}$), if present. For each coupling rate, the corresponding Q-factor is defined as $Q_{x}=\omega_{0}/\left(2\gamma_{x}\right)$, with x=T,c,v,p. From Equation (24), the maximum reflectivity is $|r_{R_{\max}}|=\gamma_{c}/\gamma_{T}=Q_{T}/Q_{c}$ and achieved at $\omega_{0}$. Therefore, it is clear that the peak reflectivity approaches unity for $Q_{c}\ll Q_{T}$. Since the waveguide can be strongly coupled to the nanocavity by reducing the distance between the two, this condition can be easily realized in practice.

The inclusion of another output port above the nanocavity, a so-called *cross-port*, is not strictly necessary, but useful if one desires to improve the laser differential quantum efficiency [40]. In fact, if the laser operates around the nanocavity resonance frequency and no cross-port is included (i.e., $\gamma_{p}=0$), the Fano mirror reflection is high and low power is coupled out to the waveguide below the nanocavity, the so-called *through-port*. In the presence of the cross-port, instead, the total quality factor of the nanocavity is reduced somewhat, but the reduction is negligible if one ensures $Q_{c}\ll Q_{v},Q_{p}$. The cross-port differential quantum efficiency is approximately proportional to $\eta_{d}=Q_{v}/\left(Q_{v}+2Q_{p}\right)$ [40] and can be easily maximized by simply ensuring $Q_{p}\ll Q_{v}$. Therefore, one obtains the practical design rule $Q_{c}\ll Q_{p}\ll Q_{v}$. Figure 13b,c shows the power reflectivity and phase of a Fano mirror with $Q_{v}=10^{5}$, $Q_{p}=1.5\times 10^{4}$ and different values of $Q_{c}$. These are typical Fano mirror parameters [97]. As the coupling between nanocavity and waveguide weakens, the peak reflectivity decreases and the mirror bandwidth shrinks. Indeed, from Equation (24) the FWHM of the power reflectivity is given by $2\gamma_{T}/\left(2\pi \right)$, thereby diminishing with decreasing $\gamma_{c}$. For the sake of convenience, $\gamma_{T}$ is denoted in the following as the Fano mirror linewidth [101].

### 5.2. Tuning Characteristics

To understand the properties of the Fano laser, it is of interest to examine the tuning characteristics of the laser, i.e., the oscillation frequency in dependence of the cavity length or nanocavity resonance frequency. This is useful both for general design purposes and more specific applications. For instance, the Fano laser can be modulated via the mirror, by dynamically changing the nanocavity resonance frequency [94,97]. The response of the threshold gain and oscillation frequency to variations of the nanocavity resonance frequency gives important indications on the on- and off-state of the laser (and transition from one to the other) in switching applications [100].

The current Fano laser models do not account for waveguide dispersion and, consequently, slow-light effects. In particular, a major assumption is that the Bloch modes traveling in the laser cavity are only coupled at the mirrors [105], thereby neglecting the gain-induced distributed feedback associated with slow-light [88]. The assumption is valid if the laser operates far from the band edge of the waveguide dispersion relation. This can be ensured by designing the nanocavity such that its resonance frequency lies far enough from the slow-light region. However, there is a number of reasons to investigate how slow-light would affect the Fano laser characteristics. As for conventional PhC lasers based on line-defect waveguides, the enhancement of the modal gain per unit length would improve the device compactness and energy efficiency. Furthermore, owing to the extreme sensitivity of the oscillation condition to variations of the cavity length and/or nanocavity resonance frequency [40,105], the additional strong coupling of the amplitude and phase conditions introduced by slow-light is likely to influence both the static and dynamic characteristics of the laser.

In the following, we solve the laser oscillation condition upon tuning of the cavity length, *L*, and nanocavity resonance frequency, $\omega_{0}$, with the right mirror reflection coefficient given by Equation (24). We follow the approach of [105] and assume that the laser oscillates at a reference frequency, $\omega_{r}$, when (1) the nanocavity resonance frequency coincides with $\omega_{r}$ and (2) the cavity length is set to a reference value, $L_{r}$. Therefore, for given $L_{r}$ and $\omega_{r}$, the left mirror phase is chosen such that a longitudinal mode lies at $\omega_{r}$ for the *cold* cavity. Then, we let either $\omega_{0}$ or *L* vary and solve for each value the oscillation condition. As a first step towards the inclusion of slow-light effects, we consider the waveguide complex propagation constant in the absence of gain-induced distributed feedback from Equation (21). Here, we Taylor-expand $k_{z}$ to first order around $\omega_{r}$ and evaluate $n_{g}$ at $\omega_{r}$, leading to

(25) $${\tilde{\beta }}_{r}\left(\omega ,N_{\mathrm{car}}\right)=k_{z}\left(\omega_{r}\right)+\frac{\omega -\omega_{r}}{c}n_{g}\left(\omega_{r}\right)-\frac{i}{2}\frac{n_{g}\left(\omega_{r}\right)}{n_{\mathrm{slab}}}\left[\Gamma_{\mathrm{FF}}\left(\omega_{r}\right)\Gamma_{y}g\left(N_{\mathrm{car}}\right)\left(1-i\alpha_{H}\right)-\alpha_{1}\right]$$

We make use of Equation (25) to model the field propagation across the waveguide. Thus, the laser oscillation condition reads $r_{L}\left(\omega \right)r_{R}\left(\omega \right)e^{+2i{\tilde{\beta }}_{r}\left(\omega ,N_{\mathrm{car}}\right)L}=1$. It should be noted that this approach obviously neglects the gain-induced distributed feedback, and employs fixed values for the group index and optical confinement factor. However, in contrast to previous studies [97,105], these values are directly computed from the dispersion relation and Bloch modes of the line-defect waveguide around $\omega_{r}$, rather than set a priori. This should provide a more quantitative basis for the estimate of the laser threshold gain. Full inclusion of slow-light effects is possible by applying a coupled-Bloch-mode approach [89], but this is beyond the scope of this paper. We assume $|r_{L}|=1$, $\alpha_{1}=5\;{\mathrm{cm}}^{-1}$ and $\alpha_{H}=1.5$. The reference wavelength, $\lambda_{r}$, is 1571nm, leading to $n_{g}\left(\omega_{r}\right)\approx 20$ and $\Gamma_{\mathrm{FF}}\left(\omega_{r}\right)\approx 62\%$. The reference cavity length is $L_{r}=8.76\;\mu m$, corresponding to 20 unit cells. The Q-factors defining the Fano mirror are $Q_{v}=10^{5}$, $Q_{p}=1.5\times 10^{4}$ and $Q_{c}=780$. The parameters of the line-defect waveguide are summarized in Table 1.

**Figure 14:**
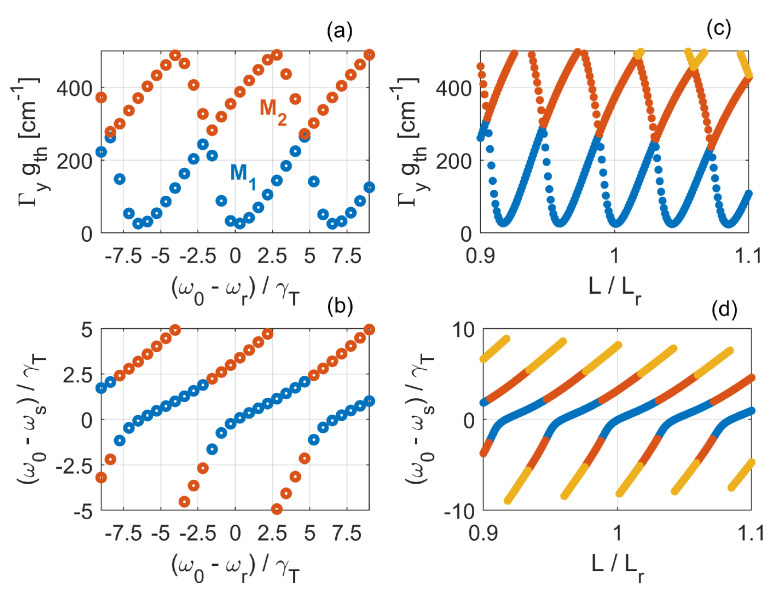
(**a**,**c**) Threshold modal gain and (**b**,**d**) effective detuning of the Fano laser upon tuning of (**a**,**b**) the nanocavity resonance frequency and (**c**,**d**) cavity length. The lasing mode, $M_{1}$, is in blue. The second and third-order modes are in red and yellow, respectively.

We start by illustrating the frequency tuning characteristics. Figure 14 shows (a) the threshold modal gain, $\Gamma_{y}g_{\mathrm{th}}$, and (b) effective detuning of $\omega_{0}$ from the oscillation frequency, $\omega_{s}$, normalized to the Fano mirror linewidth. Both the lasing mode (in blue) and second-order mode (in red) are reported. A number of features can be noted. The modal gain shows a succession of minima, occurring where $\omega_{0}$ (and thus the Fano mirror peak) aligns with a longitudinal mode of the laser cavity. The spacing of these modes is determined by the free spectral range of the cold cavity (within a small correction due to the linewidth enhancement factor). Since the group index is fixed (within the approximation of Equation (25)), the minima are equally spaced. Figure 14b confirms that the minima do correspond to zero effective detuning. The gain and effective detuning are periodic, with the same periodicity. As $\omega_{0}$ departs from a gain minimum, the threshold gain increases, because the oscillation frequency is detuned from the mirror peak. However, the gain increases more strongly in one detuning direction as compared to the other. The asymmetry is due to the non-zero linewidth enhancement factor [97].

Finally, we note that the lasing mode is always the closest to the Fano mirror peak and nearly tracks the nanocavity resonance frequency within few mirror linewidths, as evident from Figure 14b.

We now move to the length tuning characteristics. Figure 14c reports the threshold modal gain, which shows a succession of minima similarly to Figure 14a. As the cavity length is varied, the oscillation frequency has to change as well, in order to continue to fulfill the phase condition. The gain minima occur whenever the oscillation frequency aligns with the nanocavity resonance frequency, where the Fano mirror reflectivity is maximum. This is confirmed by Figure 14d, reporting the effective detuning. Again, the asymmetry in the response arises from the non-zero linewidth enhancement factor. We also note that the threshold gain at each minimum decreases with increasing cavity length, which is hard to see on the figure due to the scale. Finally, the extreme sensitivity of the response to the cavity length tuning should be emphasized [97]. A variation of 1% of *L* with respect to $L_{r}$ is enough to more than double the threshold gain as compared to one minimum. This illustrates that the mode of the Fano laser has the characteristics of a bound mode in the continuum [106,107], which was recently exploited to realize a microlaser with ultranarrow linewidth.

## 6. EDC Cavities

It is commonly held that in dielectric (non-metallic) cavities the mode volume $V_{\mathrm{eff}}\left(r_{\max}\right)$ cannot be reduced below the so-called diffraction limit [108,109], ${\left[\lambda /(2n)\right]}^{3}$, corresponding to half a guided wavelength in all three dimensions. Intuitively, this can be understood by thinking of a resonator as a rectangular box with perfect mirrors. Metallic structures supporting plasmons have been demonstrated to feature modes with much smaller volumes [110,111], but ohmic losses severely limit the Q-factor. Recently, however, new designs [8,9,10] for dielectric cavities have been proposed, which can break the diffraction limit while keeping a high Q-factor. These are so-called extreme dielectric confinement (EDC) cavities. As compared to typical PhC cavities, EDC cavities greatly enhance the electric energy density around a given point, thereby maximizing the denominator in Equation (1). The numerator essentially remains unchanged. EDC cavities are targeted at the same realm of applications where PhC cavities have flourished, such as nanolasers, nonlinear photonics and quantum technologies. In particular, a recent work [70] has pointed out that EDC cavities with few emitters embedded may be also utilized as nanolasers and nanoLEDs featuring squeezed intensity noise.

**Figure 15:**
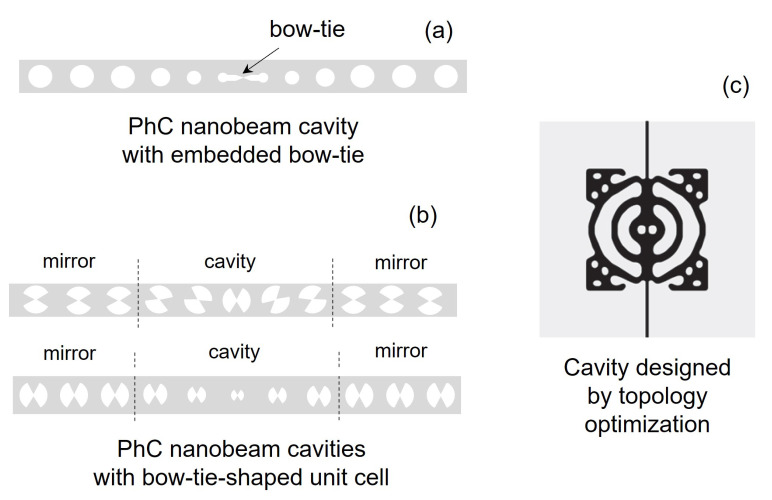
Cavities for extreme dielectric confinement (EDC). EDC cavities realized (**a**) by embedding a bow-tie in a PhC nanobeam cavity [9] and (**b**) through sub-wavelength engineering with a bow-tie shape of the unit cell of a PhC nanobeam [8,113]. (**c**) EDC cavity designed by topology optimization [10]. In Figure 15c, the gray area is air and the black area is semiconductor. Figure 15c is reprinted from [10], with the permission of AIP publishing, 2018.

Figure 15 summarizes the main design strategies of EDC cavities proposed so far. One approach, based on earlier works on air-slot waveguides and microcavities [112], is embedding a bow-tie in a PhC nanobeam cavity [9], as illustrated by Figure 15a. FDTD simulations of this EDC cavity indicate an ultrasmall mode volume $V_{\mathrm{eff}}\left(r_{\mathrm{cen}}\right)\approx 7.01\times 10^{-5}\;\lambda^{3}$ at λ≈1550nm [9], with $r_{\mathrm{cen}}$ denoting the center of the bow-tie. This structure features an air gap in the center, where a nonlinear material could be deposited. Alternatively, one may fabricate a bow-tie with connected tips and simply exploit the nonlinearities of the nanobeam material. As a result of the enhanced light–matter interaction, Kerr nonlinearities may enable a shift of one linewidth in the cavity resonant wavelength with just few photons or even a single photon (depending on the nonlinear material which is employed) [9].

As another strategy (Figure 15b), one might modify the unit cell of a PhC nanobeam by directly embedding a bow-tie into each air hole. Subsequently, a cavity is carved out of this modified nanobeam by varying, for instance, the bow-tie rotation angle [8] or size [113]. Here, a key point is that the cavity resonant mode is derived from the *air* mode of the original PhC nanobeam, i.e., the higher-frequency guided mode having most of its energy within the air holes. By including bow-ties within the holes, the mode energy density around the bow-tie tips is greatly enhanced. The variation of the bow-tie rotation angle or size misaligns the band edges of the mode dispersion relation in the cavity and mirror regions. Therefore, a cavity is formed in a very similar fashion to photonic heterostructure cavities reviewed in Section 2. The gradual variation of the bow-tie rotation angle or size ensures a gentle evolution of the resonant mode spatial envelope, thereby optimizing the Q-factor. By this approach, an EDC cavity has been experimentally demonstrated [113], with a loaded Q-factor around $10^{5}$.

It should be noted that these EDC cavity designs require minimum feature sizes (e.g., the gap between the bow-tie tips) to be on the order of few nanometres [9], which is at the limit of current fabrication techniques. In this respect, alternative designs based on topology optimization [114] may be advantageous. Topology optimization enables systematic design of EDC cavities [10] while taking into account practical constraints on the minimum manufacturable feature size [61], mainly determined by electron beam lithography or semiconductor dry etching. Essentially, the design starts from a reference structure serving as initial guess, e.g., an H1 PhC cavity or an unstructured membrane. Maxwell equations in the frequency domain are solved for a given resonant mode, excited by an electric dipole source [10]. The structure is iteratively updated by using a gradient-based optimization method, with the aim of maximizing a frequency-averaged local photonic density of states (LDOS) [115]. In fact, the LDOS scales proportionally to the ratio between Q-factor and mode volume. A constraint on the minimum feature size can be also introduced in the optimization algorithm [10]. The final result is a bow-tie surrounded by an elliptic ring grating [10] (Figure 15c). This grating effectively acts as a distributed Bragg reflector, which increases the Q-factor by reducing the in-plane radiation loss. For given targets (essentially, resonant frequency, type of resonant mode, domain size and minimum feature size), nearly identical final designs are obtained regardless of the initial guess [116], outlining the robustness of this design strategy.

**Figure 16:**
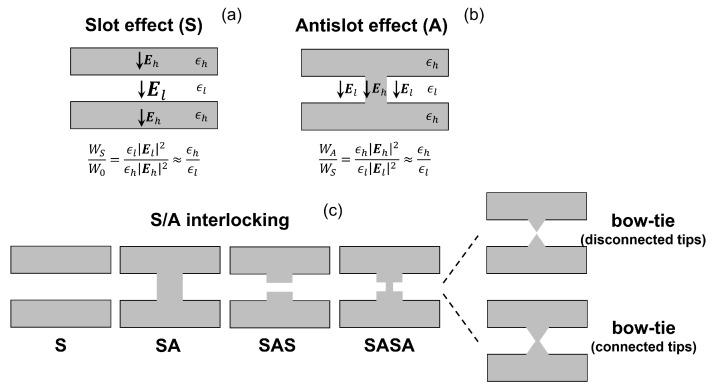
(**a**) Slot and (**b**) antislot effect to enhance the electric energy density. (**c**) Interlocking of slots and antislots leading to a bow-tie with either disconnected or connected tip.

The physical mechanisms behind the strong field concentration in a bow-tie is illustrated by Figure 16. A first mechanism is the so-called *slot effect* (Figure 16a), which is also exploited in air slot PhC cavities [112,117]. Essentially, Maxwell’s equations require the normal component of the electric displacement field to be continuous across a dielectric interface. Across a slot, the electrical permittivity, ϵ, changes abruptly from a high $\epsilon_{h}$ to low $\epsilon_{l}$ value, thereby being discontinuous. Consequently, the normal component of the electric field is forced to be discontinuous. As result, by assuming the electric field to be highly polarized in the normal direction, the electric energy density within the slot ($W_{S}$) is roughly enhanced by a factor $\epsilon_{h}/\epsilon_{l}$ as compared to that in the high refractive index medium ($W_{0}$). By building a bridge across the slot, the energy density can be further enhanced. This is the so-called *antislot effect* [8] (Figure 16b). In this case, one leverages the continuity of the tangential electric field component at the boundaries between slot and bridge (or antislot, from now on). As a result, the electric energy density within the antislot ($W_{A}$) is roughly enhanced by an additional factor $\epsilon_{h}/\epsilon_{l}$ as compared to that inside the slot. Air is conveniently used as the low permittivity medium in order to maximize the refractive index contrast. By interlocking slots (S) and antislots (A), the energy density can be progressively enhanced [9]. This strategy is illustrated by Figure 16c. The largest enhancement is achieved in the limit of a bow-tie structure. In this case, the energy density is maximum within the gap between the two tips (bow-tie with disconnected tips) or inside the tiny dielectric strip connecting them (bow-tie with connected tips), depending whether the last element of the concatenation is either a slot or antislot. As a rule of thumb, the smaller the minimum feature size is and the larger the energy density enhancement becomes [9].

## 7. Conclusions

In this paper, we have reviewed the main modal properties and design strategies of photonic crystal (PhC) cavities and cavities for extreme dielectric confinement (EDC).

In Section 2, we have provided an overview on the main properties of PhC cavities. In Section 3, we have discussed why PhC lasers are promising light sources for chip-scale optical interconnects. In Section 4, we have derived in detail the resonance condition and electric field distribution of line-defect cavities. These properties naturally follow from the spatial and frequency dependence of the Bloch modes of the line-defect waveguide on which the cavity is based. In Section 5, we have reviewed the tuning characteristics of PhC Fano lasers, with one mirror based on Fano interference. In this study, optical confinement factor and group index have been directly extracted from Bloch modes and dispersion of the line-defect waveguide. This partially accounts for slow-light and provides a more quantitative basis as compared to previous investigations. Finally, in Section 6 we have covered EDC cavities, which embed bow-tie structures to greatly enhance the electric energy density around a given point. As a result, EDC cavities squeeze the diffraction-limited mode volume of conventional PhC cavities and strengthen the light–matter interaction.

PhC and EDC cavities open up a wide range of intriguing opportunities, such as energy-efficient semiconductor lasers, nonlinear photonics and quantum electrodynamics. This paper provided an introduction to this realm of photonic cavities and their modal properties. There are many aspects left for future research, such as the limits to the light–matter interaction in these cavities and the designs of structures for particular applications.

## Figures and Tables

**Table 1:** Simulation parameters of the line-defect waveguide assumed throughout Section 4.

Parameters	Values
Lattice constant *a* [nm]	438
Slab refractive index $n_{\mathrm{slab}}$	3.17
Hole radius *r*	0.25a
Slab thickness $h_{\mathrm{slab}}$ [nm]	250
Cladding refractive index $n_{\mathrm{clad}}$	1

## Data Availability

The data presented in this study are available on request from the corresponding author.
